# The Legacy of Renato Dulbecco in the Post-Genomic Era

**DOI:** 10.3390/medicina58080974

**Published:** 2022-07-22

**Authors:** Ludovico Abenavoli, Luigi Boccuto

**Affiliations:** 1 Department of Health Sciences, University Magna Graecia, 88100 Catanzaro, Italy; 2 Healthcare Genetics Program, School of Nursing, College of Behavioral, Social and Health Sciences, Clemson University, Clemson, SC 29634, USA; lboccut@clemson.edu

The true measure of a visionary is marked by their ability to perceive and anticipate future developments in their field. It is hard to identify a subject that has witnessed a more dramatic and abrupt development in the past century than genetics. As elegantly and poignantly stated by Mukherjee, “three profoundly destabilizing ideas ricochet through the twentieth century (…): The atom, the byte, the gene” [1]. If the astonishing advancements of genetic research were able to find their way out of laboratories and into hospital units, parliaments, and even main streets, significant credit must be given to the handful of visionaries that inferred how interactions between our genome and the environment could lead to numerous multifactorial disorders—first and foremost cancer. Ten years after his departure, this issue revisits some of the many implications of the contribution of one such visionary, the Nobel Laureate Renato Dulbecco.

Dulbecco was born in 1914 in Catanzaro, the capital city of the Calabria region in the South of Italy. He was a student in the laboratory of Giuseppe Levi at the University of Turin along with two other Italian scholars who, like him, won the Nobel Prize, namely Rita Levi-Montalcini and Salvador Luria. In 1946, Dulbecco accepted Luria’s invitation to join him as a research associate at the University of Indiana at Bloomington (USA), where he shared the laboratory with another future Nobel Laureate and trailblazer in the field of genetics, James Watson. In Indiana, Dulbecco worked on the genetics of bacteriophage viruses and discovered the process of the photoreactivation of ultraviolet-irradiated bacteriophages. Subsequently, he joined the faculty at Caltech in Pasadena, California. He worked under Max Delbruck, who shared the 1969 Nobel Prize with Luria and Alfred Hershey “for their discoveries concerning the replication mechanism and the genetic structure of viruses”. At Caltech, Dulbecco studied cell transformation, in particular the ability of viruses to stimulate cellular DNA synthesis. In 1968, Dulbecco and his colleagues showed how viral DNA was capable of merging with the nuclear DNA of the host cell. He proposed that this integration would eventually lead to the addition of viral genes to the host’s genome, implying that environmental factors can induce the development of cancer by disrupting genes. In 1975, Dulbecco, along with Howard Temin and David Baltimore, was awarded the Nobel Prize in Physiology or Medicine for “discoveries concerning the interaction between tumor viruses and the genetic material of the cell” [2]. After receiving the Nobel Prize, Dulbecco decided to focus his interest on breast cancer and was a prominent promoter and advocate of the Human Genome Project. His contribution to the Project included the coordination of the Italian section, which led to the substantial coverage of the genetic sequences of the Xq24-Xqter region (the terminal portion of the long arm of the X chromosome). He continued his research and published scientific articles until his death, which occurred in 2012 at his home in La Jolla, California.

The indisputable merit of Dulbecco was paving a new way in the domain of research, expanding the area of interest in genetics from rare congenital disorders to multifactorial conditions and revealing some of the most critical pathogenic mechanisms of carcinogenesis. By arguing that the best way to understand cancer would be to sequence the human cancer genome [3,4], he not only laid the foundations of translational medicine, but he also highlighted the importance of somatic sequencing, implying a pathogenic role for somatic genetic variants—a concept vastly investigated nowadays in the studies on somatic mosaicism and late-onset disorders. This new view on genetics and genomics added a novel perspective to conventional assumptions about the relationship between genome structure and function, and between genotype and phenotype.

Dulbecco believed that science should be useful to mankind. During his Nobel lecture, he pointed out the potential dangers of environmental substances—many of which are generated by human activities—that can cause mutations leading to cancer development. He also invited the world’s governments to discourage the consumption of tobacco, a known carcinogen, but also lamented the difficulties in getting society to make the required life changes and sacrifices [5].

The articles collected in this Special Issue explore some of the many changes that Dulbecco’s vision brought to the field of medicine, from the novel and more precise approaches to the diagnosis and management of pediatric cancer to the ethical implications of the results of the Human Genome Project on the concept of “race” in healthcare; from the development of safer and more effective drugs to the profiling of candidate subjects for clinical studies. What is now defined as the post-genomic era was only possible due to the vision of scientists like Renato Dulbecco who were able to link genes and environmental factors and discover new ways to investigate the causes of multifactorial diseases and, eventually, cure them.

Incidentally, sharing the same birthplace as Dulbecco, it has been a high honor to coordinate this editorial endeavor dedicated to his legacy. It has allowed us to highlight the groundbreaking impact of his achievements and how he helped to steer the course of translational medicine and introduce genetics to popular culture. In a time before social media, he proved to be a gifted communicator, capable of sharing the most advanced innovations in a “niche” field such as genetics with the layperson. His ability to divulge even the most complex molecular notions increased his popularity beyond his illustrious scientific merits and made him a recognizable figure in the post-genomic era, where the benefits of the Human Genome Project are becoming ever more accessible and are projecting genomic sequence into the protocols of precision medicine.

Renato Dulbecco was a stimulating mentor and an example for many aspiring scholars. He valued his younger coworkers, whom he inspired and supported in many ways. A decade after his departure, his spirit lives in his work and legacy as a beacon illuminating the way for new generations of scientists.

## Data Availability

Not applicable.
